# Immunologic Role of Extracellular Vesicles and Exosomes in the Pathogenesis of Cystic Fibrosis

**Published:** 2018-02

**Authors:** Alireza Asef, Esmaeil Mortaz, Hamidreza Jamaati, Aliakbar Velayati

**Affiliations:** 1 Department of Biology. Islamic Azad University, Rasht Branch, Rasht, Iran; 2 Department of Immunology, Faculty of Medicine, Shahid Beheshti University of Medical Sciences, Tehran, Iran; 3 Clinical Tuberculosis and Epidemiology Research Center, National Research and Institute of Tuberculosis and Lung Diseases (NRITLD), Shahid Beheshti University of Medical Sciences, Tehran, Iran; 4 Chronic Respiratory Disease Research Center, NRITLD, Shahid Beheshti University of Medical Sciences, Tehran- Iran; 5 Pediatric Respiratory Disease Research Center, NRITLD, Shahid Beheshti University of Medical Sciences, Tehran- Iran

**Keywords:** Microparticles, Exosomes, sputum, Lung cystic fibrosis

## Abstract

Cystic Fibrosis (CF) is the most common lethal autosomal recessive disease that affects many organs including, lung, pancreas and liver. Cystic fibrosis is a monogenic disease and occurs in the white Caucasians. Massive neutrophil granulocyte influx in the airways is one of the characteristics of CF. Extracellular Vesicles (EVs), microvesicles, and exosomes are vesicles released from cells into extracellular space of the body and are able to influence other cells by different methods.

They have an important role in the intracellular communication by transferring information between donor and recipients cells. Granulocytes are known as the main source of microparticles in the CF patients. Microparticles derived from neutrophils are associated with the extensive neutrophil influx into airways and aggregation at the epithelial surface of the CF patient’s respiratory tract.

Exosomes are found in almost all body fluids, such as urine, sputum, Bronchoalveolar Lavage (BAL), milk, Cerebrospinal Fluid (CSF), plasma and sputum. Examination of exosomes derived from CF patients may be helpful in the characterization of pathogenesis of disease in detail. In this mini review, we have summarized the role of microparticles and exosomes in pathogenesis of CF and finally discussed the feasibility of this particle in treatment approaches.

## INTRODUCTION

Biological markers or biomarkers generally indicate the biological status, health or pathological conditions, or conditions for assessment of treatment response. Biomarkers can be used as a safe way to identify pathological conditions in various diseases, including respiratory diseases. Extracellular vesicles (EVs) and exosomes are small plasmid mucous membranes (40–150 nm) that are released from cells such as macrophages, endothelial cells, granulocytes, monocytes and lymphocytes during chemical and physical stimulation and apoptosis and are referred to as biomarkers (1,2). Exosomes are found in almost all body fluids, such as urine, sputum, Bronchoalveolar Lavage (BAL), milk, Cerebrospinal Fluid (CSF) and plasma (3,4). It contains proteins and lipids that may help in understanding its characteristics, and may represent the main source of the cells and the type of stimulation that caused its formation (5–7).

Exosomal lipids are mainly cholesterol, phospholipid, phosphatidylserine, and prostaglandin-free nuclei, mitochondrial and ribosomal proteins (8,9) that prevent enzymatic degradation of micro-RNAs in body fluids, causing a relatively long-term expression.

Persistent circulating exosomes in biological fluids can provide a vast amount of information for the pathological and physiological status (4,10, 11). Lung as an organ has a different range of cells and it is expected that exosomes have an important role in the biology of lung function as an intercellular interface in the respiratory system (12).

Besides, exosomes control the inflammation signaling through intracellular communication and as a part of response to stress, may also play a role in the respiratory tract (13,14). In this short review paper, the possible role of exosomes in the pathogenesis of cystic fibrosis has been reviewed in detail.

### Cystic fibrosis and pathogenesis

**Figure 1.**
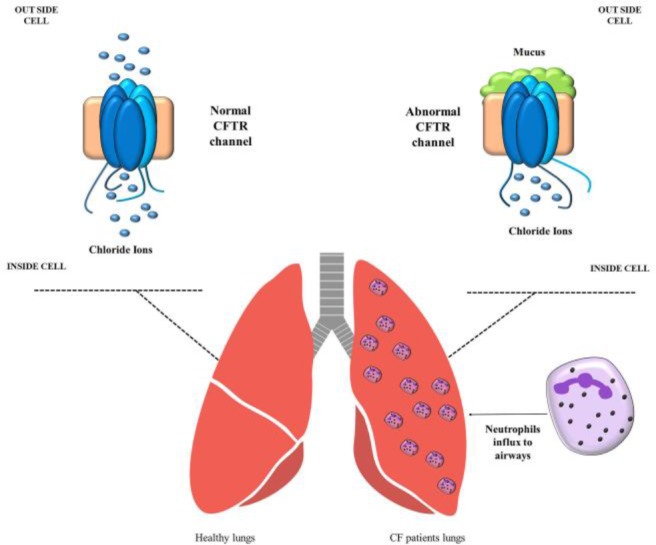
Pathogenesis of CF and neutrophils accumulation in the lungs.

CF is defined as an autosomal recessive respiratory genetic disease induced by mutations in the Cystic Fibrosis Transmembrane conductance Regulator CFTR gene, which encodes important protein in body (15). This mutation disrupts the activity of the chlorine channel and eventually causes the neutrophil influx into the airway (16), (Figure 1).

Dysfunction of chloride channel in CF patient that eventually causes neutrophil influx into the airway of CF patients.

Since the early detection of the CFTR gene mutation, 1500 different mutations have been identified for CFTR. ΔF 508 is the most common type of mutation in CF patients which is due to the deletion of phenylalanine in position 508, and is found in 66% of CF patients (14).

CFTR mutations are categorized into 6 groups based on their functional consequences:

a) Not synthesized
b) Inadequately processed
c) Not regulated
d) Abnormal conductance
e) Partially defective production
f) Accelerated degradation (17,18).

Mutations in groups a, b, and c are common mutations and mutations in groups d, e, and f are rarely observed in CF patients (15).

Defect in the CF gene leads to development of CFTR and lack of this protein, ultimately causes the unconventional transfer of chloride in the apical membrane of the epithelial cells (19,20). The results of this important defect could cause airway surface liquid depletion and induce defects in surface celiac functions and mucosal transmission (21).

Pseudomonas aeruginosa (P. aeruginosa) and Staphylococcus aureus (S. aureus) are the most common bacteria which affect CF patients (22). Emerging observations indicated that CFTR acts as a receptor for P. aeruginosa and causes intracellular uptake and bacterial destruction (23). S. aureus and P. aeruginosa are mainly located on the mucus layer of the epithelial cell in the respiratory system (24,25).

Dehydration of airway surface liquid is the main cause of impaired cilia functioning and mucociliary clearance in CF patient, and in a way causes inhaled bacteria not to be cleared from the airways (26,27).

Due to lower levels of oxygen in sputum of CF patient, P. aeruginosa can be transformed from non-mucoid to mucoid state in the respiratory system (26).

CF is characterized by high concentrations of neutrophilic chemokines such as IL-8 and accumulation of neutrophils in the airway which in turn causes activation of neutrophils (17,28). In CF patients neutrophils shows increased levels of oxidative burst, enhanced production of elastase, increased IL-8 production and decreased levels of IL-1β in airway (29–32). Moreover, due to the induction of apoptosis in the neutrophils, cells are unable to efficiently destroy the bacteria.

In CF patient, neutrophils are invaded by Pseudomonas as a main infectious agent. Neutrophils are killed by bacteria via the released proteases that prevent the adjacency of live neutrophils (33). In this way presence of large number of bacteria and their products damage tissues and lead to inflammation, tissue destruction and the creation of an environment that can lead to infection (34).

Sputum of CF patient contains inflammatory materials that could be considered as biomarkers of infectious status in the airway of CF patients (35). Sputum examination in CF patient is considered to be one of the non-invasive methods for detecting infection in the lungs (35) which contains protein and peptides that act as a biomarker for diseases and its severity (36).

Cell activation and apoptosis are known as neutrophilic features in CF patients which result in the production of microparticles that are found in the sputum of these patients (34). Studies have shown that granulocytes are commonly found in CF as a major source of microparticles.

#### Possible role of microparticles and exosomes in the pathogenesis of CF

**Figure 2.**
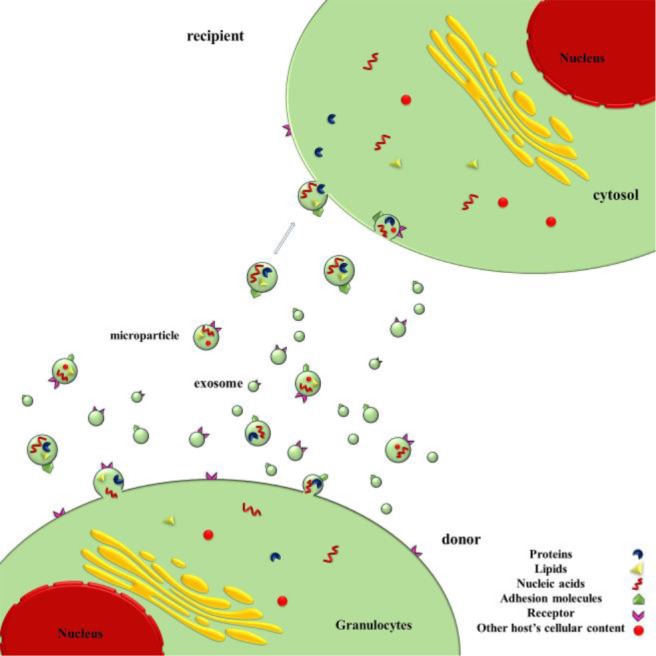
Schematic secretion of microparticles and exosomes from granulocytes to extracellular space.

Extracellular vesicles (EVs) have different subunits, such as exosomes or microvesicles that are secreted from cells into the extracellular spaces (37). The release of exosomes is done by exocytosis, while microvesicles are released by outward budding of the plasma membrane (37). EVs contains DNA, RNA, proteins and lipids which represent the cellular origin and have a specific role in inter-cellular communication (4,38), (Figure 2).

Exosomes carry the same material as a subset of EVs and are surrounded by a lipid bi-layer membrane. Exosomes show cup-like morphology when seen under the Transmission Electron Microscopy (TEM), (39–41). Exosomes (less than 150 nm) are vesicles that are released to the extracellular environment. Recent data show that exosomes are an alternative way of eliminating waste products in order to maintain cellular homeostasis (4,42–45) and were shown to have immune regulatory effects (46,47). For exosomes to be released, several cellular steps must be completed including Intraluminal Vesicles formation (ILVs) in Microvesicles bodies (MVBs), transferring of MVBs to the plasma membrane and fusion of MVBs with the plasma membrane (48–50), (Figure 2). Recent observations show that microparticles can divert different messages between cells and can change the target cell biology in a variety of ways such as stimulating other cells by surface-expressed ligands, acting as a signaling complex (45), and transmission of surface receptors from one cell to another. Transmission of proteins, bioactive lipids, mRNA and miRNA into target cells (45) can also transmit infectious components (45).

It has been shown that microparticles are derived from CF granulocytes that are associated with extensive entry of neutrophils into airways and aggregation at the epithelial surface of the respiratory tract in CF patients (51).

Microparticles cause persistence of inflammation and play a role in the pre-inflammatory response of CF patients (2). Extracellular vesicles participate in normal biological processes such as:

a) Tissue repair (52)
b) Immune surveillance (53)
c) Blood coagulation (54)
d) Stem cell maintenance (55)

by delivering effectors such as transcription factors, small and large noncoding regulatory RNAs such as mRNAs (56).

Extracellular vesicles are mediators for intercellular communication and transfer protein, lipid and nucleic acid (53,57, 58) and have an association with tumorigenesis, Alzheimer’s and Parkinson’s diseases (59–62).

Bronchial epithelial cells produce exosomes in CF patients which suggest that exosomes obtained from biofluid of CF patients are a non-invasive biomarker for the disease (9).

Prolyl Endopeptidase (PE), which is necessary for the production of the neutrophil chemo-attractant tripeptide Pro-Gly-Pro (PGP) from collagen, is released by the respiratory tract exosomes and plays a role in regeneration and inflammation of respiratory system (63). This data has been confirmed by observation of sputum exosomes of CF patient with bacterial infection, who have an elevated level of PE (42).

Evidence suggests that epithelial cells in CF patients, frequently release vesicles with particular size especially into the airways which can be useful in identifying the disease status (43). Besides, the amount and type of mucin on the surface of the exosome indicates the exosome size and will be an alert in CF patients. Due to functional impairment in the CFTR, exosomes can be useful for reconstructing its function (64).

Studies on CF patient’s exosomes shows that microvesicles and exosomes are potentially useful as vectors for transferring CFTR and can help with the functional correction of human CF cells (65).

**Figure 3.**
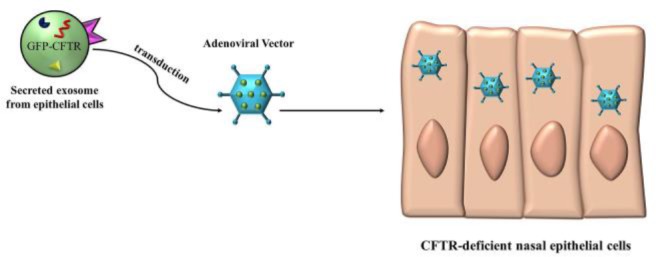
Ongoing projects on overexpression of CFTR gene in deficient gene

Recently a lot of research has been done for the reconstruction of CFTR receptor on the defect cells and suggests that using exosomes may help to cure this disease. For example exosomes derived from the human cell line A549 (adenocarcinomal human alveolar basal epithelial cells) and calu-3 (epithelial cell), transduced with an adenoviral vector overexpressing a GFP-tagged CFTR (GFP-CFTR), were able to deliver the GFP-CFTR glycoprotein and mRNA (GFP CFTR) to CFTR-deficient nasal epithelial cells and restore CFTR function in a dose-dependent manner (41) (Figure 3).

Secreted exosomes from epithelial cells transduced with an adenoviral vector overexpressing a GFP-CFTR and deliver the GFP-CFTR glycoprotein and mRNA (GFP CFTR) to CFTR-deficient nasal epithelial cells and restore CFTR function (65).

## CONCLUSION AND FUTURE PERSPECTIVES

Exosomes are thought to be particles that cause cell-cell communication, are found in various body fluids and are able to exchange a variety of information between cells.

We can understand the status of various diseases like CF by studying exosomes and their contents. On the other hand, exosomes are known as biomarkers for the diagnosis of disease and have the ability to be used as vectors to restore the function of the CFTR in CFTR deficient patients, and may help to treat CF.

Besides, blocking microparticles such as exosomes may exacerbate the disease and promote immunity-enhancing induced by particles, and could be considered as a suggestive approach to treat CF diseases.
